# A Simple Interfacial Platform for Homogeneous Electrochemical Immunoassays Using a Poly(Vinylimidazole)-Modified Electrode

**DOI:** 10.3390/s17010054

**Published:** 2016-12-29

**Authors:** Young-Bong Choi, Won-Yong Jeon, Hyug-Han Kim

**Affiliations:** 1 Department of Chemistry, College of Natural Science, Dankook University, Anseo-Dong, Cheonan, Chungnam 330-714, Korea; chem0404@dankook.ac.kr; 2 Department of Nanobiomedical Sciences and BK21 PLUS NBM Global Research Center for Regenerative Medicine, Dankook University, Anseo-Dong, Cheonan, Chungnam 330-714, Korea; powerwy@hanmail.net

**Keywords:** immunoassay, hippuric acid, electrochemical biosensor, nickel ion, mediator

## Abstract

In this study, a homogeneous method featuring simple, one-step detection was developed to analyze hippuric acid (HA), a major metabolite of toluene. High sensitivity was achieved with the facile immobilization of poly(vinylimidazole) (PVI) on an indium tin oxide (ITO) electrode. Using a previously developed approach, pentacyanoferrate was coordinated with pyridyl-*N* ligands, and the redox-active Fe(II/III) centers were bound to Ni(II) ions on the electrode via electrostatic cyanide bridges. The detection was accomplished by the competitive binding of free HA and pentacyanoferrate-(4-aminomethylpyridine-hippuric acid) (Fe-HA, the electron transfer mediator) to the HA antibody on the Ni(II) ions-modified PVI-ITO (Ni-PVI-ITO) electrode. The electrical and physicochemical characterization of the electrode was carried out by cyclic voltammetry, differential pulse voltammetry, field emission scanning electron microscopy, and X-ray photoelectron spectroscopy. At low mediator concentrations, the electrical signals were proportional to the HA concentration between 0.1 µg/mL and 1.0 mg/mL. The same method may be extended to other small organic molecules.

## 1. Introduction

Many studies of hexacyanoferrate(III) as ferricyanide mixed with Ni(II) ions have been carried out because of its good reversible redox properties and zeolitic structure [1]. It has been reported that hexacyanoferrate can attach to Ni(II) ions through its cyanide bridges [2,3]. Ni(II) ions-mixed ferricyanide has been used extensively in glucose sensing [4], ascorbic acid sensing [5], uric acid sensing [6], determining thiosulfate [7], and potassium ion sensing [8]. Moreover, it has an excellent electrocatalytic activity for enzyme oxidation [9]. Ni(II) ions-modified ferricyanide has a characteristic electrical behavior with a redox potential of 0.46 V (versus Ag/AgCl) [10].

Metal ions such as Ni(II), Cu(II), and Zn(II) are known to bind to the nitrogen atoms of imidazole and pyridine rings [11,12]. The nitrogen of an imidazole ring has been bound to metal ions and metal complexes such as biological ligands [13]. Ferricyanide redox complex has been studied using both electrochemical and spectroscopic techniques [14]. Moreover, the ferricyanide redox complex has been used for the determination of various target substrates because of its reversible redox system [15,16]. Pentacyanoamminferrate(III) which has five cyanide groups and one amine complexed with an Fe(III) ion, has a similar structure and electrical characterictics as the ferricyanide redox complex, therefore a small molecule having imidazole and pyridine rings can be replaced by the amine group of pentacyanoamminferrate [17,18].

Toluene, a commonly known industrial organic solvent, is a clear, colorless, water-insoluble liquid with a distinctive smell. Toluene is widely used as a solvent in the petroleum industry and in paints, thinners, detergents, adhesives, and many other products [19]. Exposure to toluene mostly occurs by inhalation, however, toluene can be absorbed to a lesser degree through ingestion and the skin. It is distributed quickly to highly perfused tissues such as the brain and liver, with an accumulation in tissues that have high lipid content. A high concentration of toluene can cause headaches, fatigue, dizziness, nausea, loss of consciousness and even death from respiratory failure or arrhythmias [20]. For these reasons, the quantitative determination of human body toluene exposure is important. Hippuric acid (HA) is a major toluene metabolite. Urinary HA concentration is widely measured as a “target compound of toluene exposure” [21]. Ultraviolet visible spectroscopy (UV-vis) [22], gas chromatography (GC) [23], and high performance liquid chromatography (HPLC) [24] have all been used for the determination of HA. An electrochemical immunoassay method in particular has been applied to measure HA concentrations because this method involves simple, relatively low cost instrumentation with miniaturization ability, portability, disposability, and full automation [25,26,27].

Electrochemical immunoassays can be divided into homogeneous and heterogeneous methods, depending on whether the antibody or antigen needs to be immobilized on the electrode or not. Most reported electrochemical immunoassays have used heterogeneous methods, which usually display higher sensitivity and less interference [28,29,30], however, it has been reported that the physical adsorption and covalent conjugation methods used for antibody immobilization on a solid surface can cause random orientation, denaturation, and steric hindrance in the antibodies. The latter reduces the specificity and reproducibility during the immunoassays [31]. In comparison, homogeneous electrochemical immunoassays are relatively simple, inexpensive, and can be performed in a single step without the need for separation [32].

In our previous paper, Ni(II) ions were bound to a polymer film electro-polymerized on an electrode, for the electrochemical immunoanalysis of HA using Fe as an electron transfer mediator [18]. The objective of the present study was to develop a simple electro-polymerized poly(vinylimidazole) (PVI) polymer film on an indium tin oxide (ITO) electrode that also enables HA detection with high sensitivity in a homogeneous electrochemical immunoassay. Optimized conditions were found for forming the PVI polymer layer on the electrode by cyclic voltammetry (CV). Then Ni(II) ions were bound to the polymer due to their high affinity for the many imidazole rings in the PVI. The as-prepared Ni-PVI-ITO electrode was used to measure low HA concentrations with Fe-HA and HA antibody (*anti*-HA). In the Ni(II) ions-based electrochemical immunoassay, high sensitivity was shown at low mediator concentrations.

## 2. Materials and Methods

### 2.1. Chemicals and Reagents

Ammonium disodium pentacyanoamminferrate(III) dehydrate was purchased from Fluka (Buchs, Austria). Monoclonal *anti*-HA was donated by HBI (Seoul, Korea). 4-Aminomethylpyridine, Ni(II) chloride (NiCl_2_), buffering salts, HA, 1-vinylimidazole, azobisisobutyronitrile (AIBN), 2-ethoxy-1-ethoxycarbonyl-1,2-dihydroquinoline (EEDQ), and other chemicals were purchased from Sigma-Aldrich Co. (Milwaukee, WI, USA). Phosphate buffered saline (PBS, 4.3 mm NaH_2_PO_4_, 15.1 mM Na_2_HPO_4_, and 140 mM NaCl) and all other solutions were prepared using deionized Milli-Q water (Milli-Q^®^ Acdemic, Molsheim, France). PVI and [Fe(CN)_5_(amp-HA)]^3−/2−^ (Fe-HA) were prepared by adapting the previously reported procedures [17,18,33]. Analytical grade reagents were used without further purification.

### 2.2. Apparatus and Electrodes

The morphology of the PVI-electro-polymerized films was demonstrated using FE-SEM (Hitachi S-4300, Tokyo, Japan). Also, the immobilized Ni(II) ions on the PVI-electro-polymerized ITO (Ni-PVI-ITO) electrode was investigated by XPS (SPECS, Berlin, Germany) at 12 kV and 3 mA current. Electrochemical measurements were carried out in a Faraday cage with a model 660B Electrochemical Workstation (CH Instruments, Austin, TX, USA) interfaced to a computer. The electrochemical characteristics of Fe-HA were studied using 6.0 mm diameter ITO electrodes as the working electrodes. An Ag/AgCl micro reference electrode (3.0 M KCl, Cypress, Lawrence, KS, USA) scrolled with a 0.5 mm diameter platinum wire counter electrode was used. CV and DPV analyses were conducted with 40 μL of Fe-HA 0.05 mM dissolved in 0.1 M PBS buffer (pH 7.4). The high performance liquid chromatography (HPLC) data was measured by CTS-30 (Younglin, Anyang, Korea) equipped with a C-18 column (Waters, Sunfire, Ireland) using methanol, DI water, and acetic acid (ratio is 7:3:0.1) as eluent. The absorbance of HA was observed at 16 min.

### 2.3. Immobilization of PVI and Nickel(II) Ions on ITO Electrode

**Figure 1:**
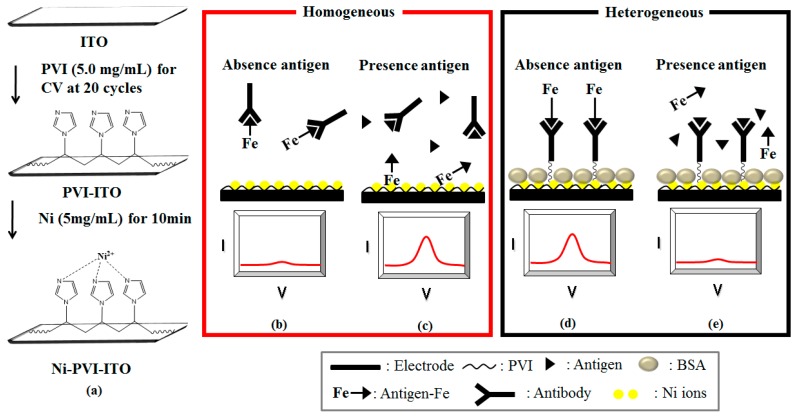
Schematic of: (**a**) electrode preparation and the immunoreactions (homogeneous and heterogeneous in the absence (**b**,**d**); and presence of antigen (**c**,**e**)). The homogeneous assay mode was used in this paper.

The ITO electrodes were cleaned by immersion in piranha solution (H_2_O_2_:H_2_SO_4_ = 3:1, *v*:*v*) for 5 min, and then washed with deionized (DI) water. The washed ITO electrode was dried completely in a nitrogen gas stream at room temperature after ultrasonication for 5 min in DI water. In a typical process, 40 μL of PVI solution in PBS (5.0 mg/mL) was electro-polymerized on the electrodes by CV for 20 cycles from −0.5 to 1.0 V at a scan rate of 0.1 V/s. After cleaning with DI water, the PVI-electro-polymerized ITO electrodes were loaded with NiCl_2_ solution (5.0 mg/mL) for 30 min, and washed again with DI water. Finally, 40 μL of Fe-HA solution (0.05 mM) was allowed to react on the Ni-PVI-ITO electrodes for 10 min, and the reaction was carried out by CV in 1× PBS solution after washing (Figure 1a).

### 2.4. Optimization of Electrode Modification Procedures

For the immobilization of PVI on the ITO electrode, CV was carried out using 5.0 mg/mL PVI solution for 2, 4, 6, 8, 10, 20, and 30 cycles. The process was also tested using different PVI concentrations (0.1, 0.5, 1.0, 5.0, 10.0, and 15.0 mg/mL) for 20 cycles each. NiCl_2_ solution of various concentrations (0.1, 0.5, 1.0, 5.0, 10.0, 20.0, and 50.0 mg/mL) and pH values (5, 6, 7, 8, 9, 10, and 11) were used for loading Ni(II) ions onto the PVI electro-polymerized ITO electrode, and the loading time was 5, 10, 20, 30, and 60 min. Finally, CV data were collected as Fe-HA (40 μL of 0.05 mM solution) was absorbed onto the modified ITO electrode.

### 2.5. Electrochemical Measurements

In this work, a homogeneous method was used for detecting HA. The mechanisms of the homogeneous and heterogeneous methods are presented in Figure 1b–e. The feasibility of the HA immunoassay was investigated according to the following two steps. (1) Fe-HA antigen sample (40 μL, 0.05 mM) was mixed with various concentrations of *anti*-HA, and placed into a microtube for 20 min at room temperature; and (2) CV data were collected from −0.5 to 1.0 V versus Ag/AgCl with a scan rate of 0.1 V/s, and plotted as the electrochemical immunosensor signal. The competitive immunoassay of HA was carried out as follows; (1) Fe-HA antigen (40 μL, 0.05 mM) and *anti*-HA (40 μL, 0.6 mg/mL) were mixed with either HA solution or human urine matrix in the microtube, and incubated for 20 min at room temperature; and (2) afterwards, the mixed solution was measured electrochemically by DPV on the Ni-PVI-ITO electrode from −0.2 to 0.8 V versus Ag/AgCl, with a pulse amplitude of 0.05 V and a pulse width of 50 ms. A spike test of HA in a urine sample was also performed to demonstrate the feasibility of our method in the presence of a human urine matrix. In the spike test, the electrochemical immunoassay was conducted with mixed urine solutions (40 μL) containing Fe-HA antigen (0.05 mM) and spiked HA in the range of 0.001 to 1.0 mg/mL in human urine. All baselines of CV and DPV were corrected by the CHI 660B software.

## 3. Results and Discussion

### 3.1. Morphology of PVI-Electro-Polymerized ITO Electrode

**Figure 2:**
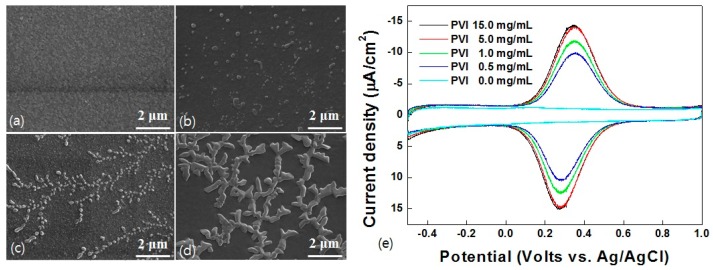
FE-SEM images (×30 K) on (**a**) bare ITO electrode and electro-polymerized ITO electrode with PVI concentrations of (**b**) 0.5 mg/mL; (**c**) 1.0 mg/mL; and (**d**) 5.0 mg/mL. (**e**) Cyclic voltammograms of Fe-HA (50 μM) based on Ni-PVI-ITO electrode which PVI concentrations of 0.0, 0.5, 1.0, 5.0, and 15.0 mg/mL.

The surface morphology of the PVI-electro-polymerized ITO electrode was observed by FE-SEM. Images of the ITO electrode in pristine condition and after electro-polymerization in PVI solutions (0.1, 1.0, and 5.0 mg/mL) are shown in Figure 2a–d, respectively. At 1.0 mg/mL, a significant area on the ITO electrode was covered by leaf-shaped PVI film, and a thicker film was clearly created with the 5.0 mg/mL PVI solution. The cyclic voltammograms in Figure 2e show that a maximum saturated current of −13.8 μA was reached with 5.0 mg/mL PVI, and it did not increase when a more concentrated solution (15.0 mg/mL) was used. Therefore, the 5.0 mg/mL PVI solution could saturate the current and produce a sufficient amount of PVI deposit to react with Fe-HA.

### 3.2. Optimization of Ni-Modified PVI-ITO Electrode Fabrication

**Figure A1:**
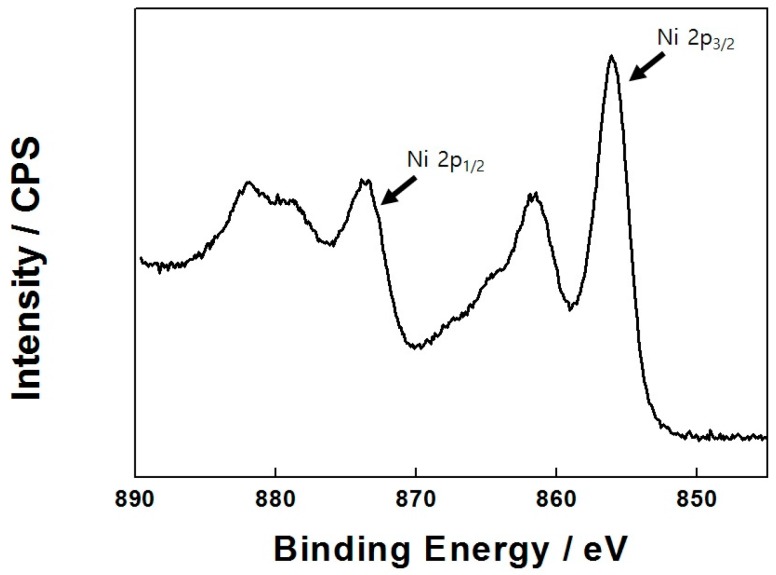
X-ray photoelectron spectrum of Ni(II) ions-modified PVI-ITO electrode.

The identified optimal conditions for fabricating the Ni-PVI-ITO electrode for reacting with Fe-HA were; 5.0 mg/mL PVI solution (as discussed above), 20 electro-polymerization cycles, 5.0 mg/mL NiCl_2_, pH = 7, and 30 min reaction time. Ni(II) ions immobilized onto the PVI-ITO electrode were investigated by XPS at 12 kV and 3 mA (Figure A1). The results show that the main Ni(II) peak appeared in 856.08 eV. This XPS data is almost same as reported in a previous paper [34].

### 3.3. Electrochemical Characterization of Nickel(II) Ions Modified PVI-ITO Electrode

**Figure 3:**
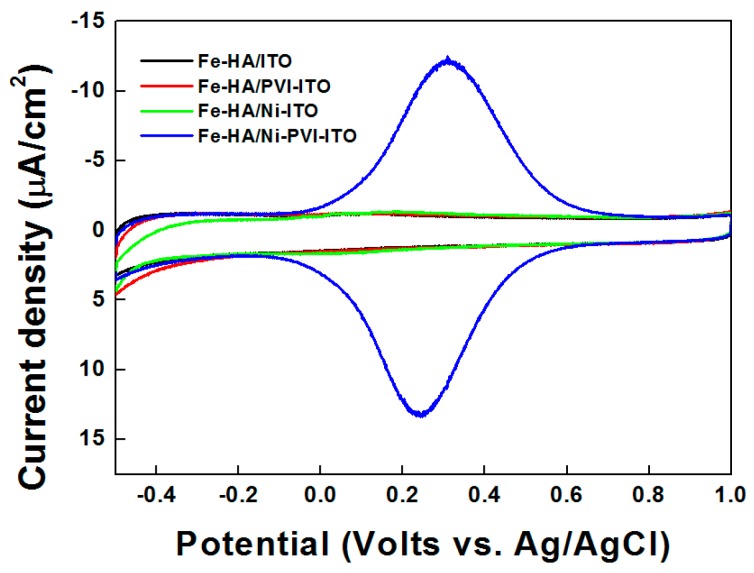
Cyclic voltammograms of 50 μM Fe-HA reacted on bare ITO (black line), PVI-ITO (red line), Ni-ITO (green line), and Ni-PVI-ITO (blue line) electrode for 10 min.

The cyclic voltammograms of Fe-HA reacted on ITO (black line), PVI-ITO (red line), Ni-ITO (green line), and Ni-PVI-ITO (blue line) electrodes are shown in Figure 3. Reversible redox behavior of the reacted Fe-HA was only observed on the Ni-PVI-ITO electrode (blue line), with a peak separation (ΔE) of 60 mV occurred at 0.1 V/s scan rate. No Fe-HA peaks were observed in the other samples, indicating that the immobilized Ni(II) ions are necessary for reaction with the cyanide groups of Fe-HA [2,3]. In other words, Ni(II) ions cannot modify ITO electrode without PVI. Finally, Fe-HA cannot bind to the ITO electrode without any substrates such as PVI and Ni(II) ions.

### 3.4. Immune Reaction between Anti-HA and Fe-HA 

**Figure 4:**
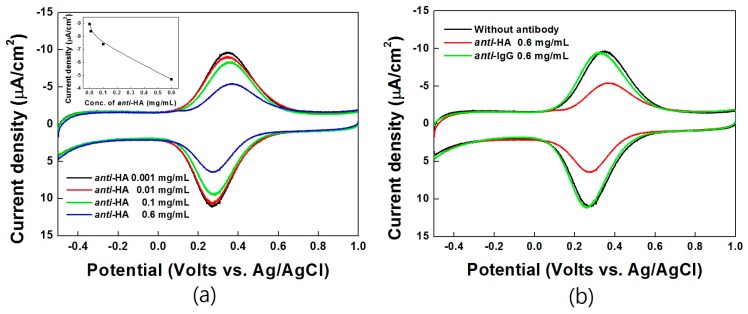
Cyclic voltammograms of Fe-HA (50 μM) reacted Ni-PVI-ITO electrode with (**a**) *anti*-HA (concentration range of 0.001 mg/mL (black line), 0.01 mg/mL (red line), 0.1 mg/mL (green line), and 0.6 mg/mL (blue line)) and (**b**) *anti*-IgG for 10 min. Inset: concentration curve of *anti*-HA at CV of 0.35 V.

Figure 4 show the CV data for the HA electrochemical immunoassay using the optimized Ni-PVI-ITO electrode, after reacting with *anti*-HA of different concentrations (0.001, 0.01, 0.1, and 0.6 mg/mL) and *anti*-IgG (0.6 mg/mL) in a microtube. 

Fe-HA was used as a redox mediator that transfers electrons to the electrode, and the HA antigen could be tagged with *anti*-HA in the immune reaction. The corresponding calibration curve is shown in the inset of Figure 4a, in which the electrical current decreases linearly with increasing *anti*-HA concentration from 0.001 to 0.6 mg/mL. These results indicate that Fe-HA binds strongly to *anti*-HA, and the bound complex could not be absorbed on the Ni-PVI-ITO electrode due to the large, heavy *anti*-HA. *Anti-*IgG was also tested as control antibody to compare with *anti*-HA (Figure 4b). The results show that only *anti*-HA shows an immune reaction response with Fe-HA under the same conditions.

### 3.5. Competition with HA and Fe-HA 

**Figure 5:**
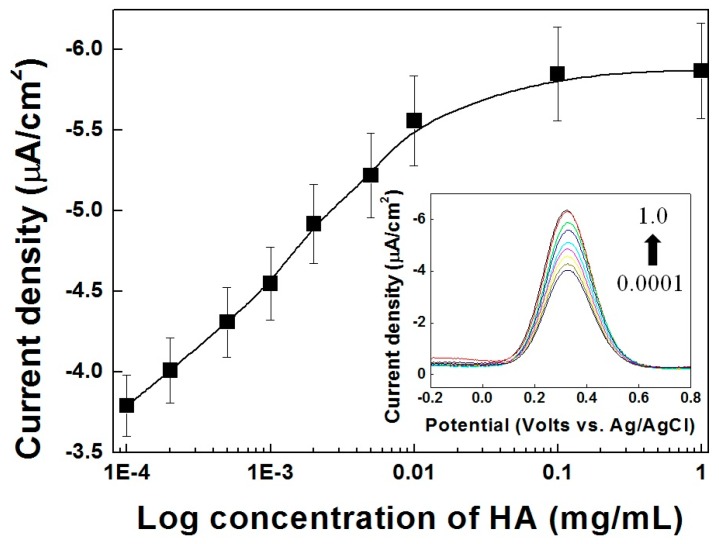
Differential pulse voltammetry vs. concentration curves of Fe-HA (50 μM) reacted with variable HA concentrations ranging from 0.0001 to 1.0 mg/mL at 0.35 V. Inset: DPV of Fe-HA (50 μM) with variable HA concentrations ranging from 0.0001 to 1.0 mg/mL.

The calibration curves for the HA electrical immunoassay with increasing HA antigen concentration are presented in Figure 5. Both free HA and Fe-HA can bind to *anti*-HA, therefore the competitive immunoassay with Fe-HA showed typical DPV curves from low to high HA concentration (inset of Figure 5). As mentioned above, the binding to the large *anti*-HA molecule obstructs the migration of Fe-HA the solution to the Ni-PVI-ITO electrode. In addition, it has been reported that the small Fe-HA antigen is more competitive than the linked one [35]. As shown in Figure 5, the magnitude of the anodic current (i_p,a_) at 0.35 V versus Ag/AgCl was chosen to represent the concentration of HA. The value of i_p,a_ was found to be proportional to the HA concentration in the log scale detection range of 0.0001 to 0.01 mg/mL, with a limit of detection (LOD) of 4.15 μA (*n* = 4 is the number of different electrodes used). Since the cutoff concentration of HA in human urine samples is 2.0 mg/mL, this electrochemical immunoassay is sufficient for detecting HA in clinical diagnosis [19].

### 3.6. Electrochemical Detection of HA-Spiked Urine Samples

**Figure 6:**
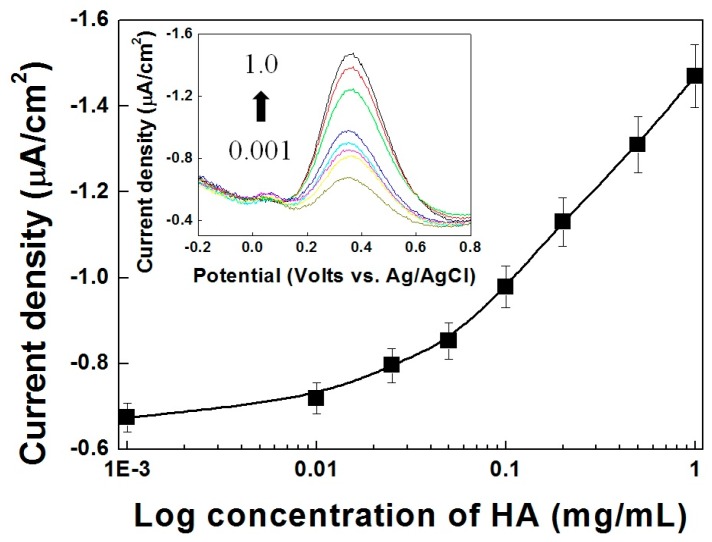
Concentration curve of differential pulse voltammetry reacted Fe-HA (50 μM) with variable HA spiked urine sample concentration ranging from 0.001 to 1.0 mg/mL at 0.35 V. Inset: DPV of Fe-HA (50 μM) with variable HA concentration ranging from 0.001 to 1.0 mg/mL.

To demonstrate the practical application of the Ni-PVI-ITO electrode, we spiked various concentrations of HA into a human urine sample. Figure 6 shows the calibration curves of the electrical immunoassay with Fe-HA for different HA concentration. In the pure PBS buffer system, the Ni-PVI-ITO electrode showed a reversible redox response to the Fe-HA moieties (as shown in Figure 5). In the human urine sample, however, the slope of the curve increased dramatically with increasing HA concentration. Additionally, the electrical anodic currents in the inset of Figure 6 indicate weaker signals in the urine sample than in the pure PBS buffer system, since the former contains many interfering substrates such as ascorbic acid, uric acid, dopamine, and urea. Nevertheless, the value of i_p,a_ in HA spiked urine was found proportional to the HA concentration in the log scale detection range of 0.05 to 1.0 mg/mL, with a LOD of 0.788 μA (*n* = 4), so with our Ni-PVI-ITO electrode system, Fe-HA still shows an excellent response as a reversible redox mediator. All recently electrochemical HA detection papers are presented in Table 1. The concentration in HA spiked urine were compared with HPLC data (Table A1). Therefore, this Ni(II) ion-based electrical immunoassay shows high sensitivity in both PBS and urine with a low mediator concentration compare to other papers. 

## 4. Conclusions

In the present study, a PVI-electro-polymerized ITO electrode with immobilized Ni(II) ions was developed for the homogeneous electrochemical measurement of hippuric acid (HA), a known metabolite of toluene. The resulting Ni-PVI-ITO electrode shows high electrostatic affinity for the free hippuric acid-labeled mediator (Fe-HA) without binding to the antibody (*anti*-HA). This method demonstrated a simple design, high sensitivity and selectivity, and the ability to detect HA in physiological systems. Our homogeneous electrochemical immunoassay shows that antigens bearing small molecules can be detected with high sensitivity. This fabrication strategy also has considerable potential for healthcare applications such as point-of-care testing (POCT), when other biomarkers are conjugated with Fe(II/III) mediators.

## Figures and Tables

**Table 1:** A comparison with previous papers about electrochemical immunosensing of HA.

Reference	Concentration of Antigen (HA) (mg/mL)	Mediator	Amount of Mediator (mg/mL)	Urine Test (mg/mL)
Our study	0.0001 mg/mL	Fe(CN)_5_(amp-HA)	0.02276	0.001
[18]	0.01 mg/mL	Fe(CN)_5_(amp-HA)	2.276	N/A*
[31]	0.01 mg/mL	Os(dmo-bpy)_2_(amp-HA)Cl	0.015	0.01
[17]	0.001 mg/mL	Fe(CN)_5_(amp-HA)	0.15	0.001
[28]	0.1 mg/mL	Os(phen)_2_(amp-HA)Cl	1.0	0.1
[30]	0.1 mg/mL	Os(dme-bpy)_2_(amp-HA)Cl	2.0	N/A*
[36]	10 mg/mL	Ferrocence-HA-Lysine	1.0	10

* N/A: method was not applied to a urine sample.

## Appendix A

**Table A1:** Determination of HA in urine by HPLC and our sensor method (*n* = 4).

Concentration of HA (mg/mL)	This Sensor Method (mg/mL)	HPLC (ppm)	RSD (%)
0.5	0.49 ± 0.024	499.74	1.75
1.0	0.98 ± 0.049	999.25	1.93
